# The neutrophil–lymphocyte ratio and its utilisation for the management of cancer patients in early clinical trials

**DOI:** 10.1038/bjc.2015.67

**Published:** 2015-02-26

**Authors:** R Kumar, E Geuna, V Michalarea, M Guardascione, U Naumann, D Lorente, S B Kaye, J S de Bono

**Affiliations:** 1 The Institute of Cancer Research and the Royal Marsden Hospital, Downs Road, Sutton, Surrey, London SM2 5PT, UK; 2 The Institute of Cancer Research, 15 Cotswold Road, Downs Road, Sutton, Surrey, London SM2 5NG, UK

**Keywords:** prognostic score, phase 1 trial, inflammation, neutrophil–lymphocyte ratio, survival, RMH score

## Abstract

**Background:**

Inflammation is critical to the pathogenesis and progression of cancer, with a high neutrophil–lymphocyte ratio (NLR) associated with poor prognosis. The utility of studying NLR in early clinical trials is unknown.

**Methods:**

This retrospective study evaluated 1300 patients treated in phase 1 clinical trials between July 2004 and February 2014 at the Royal Marsden Hospital (RMH), UK. Data were collected on patient characteristics and baseline laboratory parameters.

**Results:**

The test cohort recruited 300 patients; 53% were female, 35% ECOG 0 and 64% ECOG 1. RMH score was 0–1 in 66% and 2–3 in 34%. The median NLR was 3.08 (IQR 2.06–4.49). Median OS for the NLR quartiles was 10.5 months for quartile-1, 10.3 months for quartile-2, 7.9 months for quartile-3 and 6.5 months for quartile-4 (*P*<0.0001). Univariate analysis identified RMH score (HR=0.55, *P*<0.0001), ECOG (HR=0.62, *P*=0.002) and neutrophils (HR=0.65, *P*=0.003) to be associated with OS. In multivariate analysis, adjusting for RMH score, ECOG, neutrophils and tumour type, NLR remained significantly associated with OS (*P*=0.002), with no association with therapeutic steroid use. These results were validated in a further 1000 cancer patients. In the validation cohort, NLR was able to discriminate for OS (*P*=0.004), as was the RMH score. This was further improved on in the RMH score+NLR50 and RMH score+Log_10_NLR models, with an optimal NLR cutoff of 3.0.

**Conclusions:**

NLR is a validated independent prognostic factor for OS in patients treated in phase 1 trials. Combining the NLR with the RMH score improves the discriminating ability for OS.

Phase 1 oncology clinical trials are dose- and toxicity-finding studies for novel compounds or combinations that will potentially be used for evaluation in future trials. These are generally tested in patients with advanced cancer who have exhausted standard care options. The likely benefit from these agents may be limited and the commitment from the patient is significant. Predicting which patients will benefit from a phase 1 clinical trial is challenging, as their general health may be declining with advancing disease, and they may experience toxicity in exchange for limited benefit (Roberts *et al*, 2004).

To assist with clinical decision-making and patient selection, several prognostic models have been developed that can be applied at the bedside (Chau *et al*, 2011; Fussenich *et al*, 2011; Olmos *et al*, 2011; Ploquin *et al*, 2012). The Penel model for 90-day mortality (Penel *et al*, 2010), the Hammersmith score for OS (Stavraka *et al*, 2014), and the Royal Marsden Hospital (RMH) score for OS (Arkenau *et al*, 2009) are the only models that have been validated in the phase 1 population. The RMH score is currently used in the Drug Development Unit, RMH. This score comprises three components, each assigned 1 point: albumin <35 g l^−1^, lactate dehydrogenase (LDH) >upper limit of normal, and >2 sites of metastases. Patients scoring 0–1 have a median OS of 33.0 weeks, whereas those scoring 2–3 have an inferior median OS of 15.7 weeks.

Cancer-related inflammation is the seventh hallmark of cancer (Hanahan and Weinberg, 2011), with inflammatory cells and mediators being an essential component of the tumour microenvironment. This inflammatory response is detectable in the peripheral blood, evidenced by neutrophilia and/or lymphopenia (Mantovani *et al*, 2008). Moreover, the neutrophil–lymphocyte ratio (NLR), derived from the quotient of the absolute neutrophil count and the absolute lymphocyte count, is prognostic for patient outcomes in a variety of tumours (Guthrie *et al*, 2013; Templeton *et al*, 2014). A high NLR has been shown to be an independent prognostic factor in many advanced cancers with varying thresholds of NLR defined as being significant, including colorectal cancer (NLR>5) (Walsh *et al*, 2005; Kishi *et al*, 2009), advanced gastric cancer (NLR⩾2.5) (Yamanaka *et al*, 2007), advanced pancreatic cancer (NLR>5) (An *et al*, 2010), castration-resistant prostate cancer (NLR>3) (Keizman *et al*, 2012a), metastatic renal cell carcinoma (NLR⩾3) (Keizman *et al*, 2012b; Pichler *et al*, 2013), nasopharyngeal carcinoma (NLR>2.5) (Chang *et al*, 2013), non-small cell lung cancer (Sarraf *et al*, 2009), malignant mesothelioma (NLR⩾5) (Kao *et al*, 2010), advanced cervical cancer (NLR⩾1.9) (Lee *et al*, 2012) and advanced ovarian cancer (NLR⩾2.60) (Cho *et al*, 2009). This may be particularly relevant in the development of drugs targeting the immune checkpoint, such as CTLA-4 (cytotoxic T-lymphocyte-antigen-4) and PD-1 (programmed cell death 1)/PD-L1 (programmed death-ligand 1) targeting antibodies.

The prognostic utility of the NLR, a marker of systemic inflammation, for patients with advanced cancer entering phase 1 trials have not been explored. In this study, we hypothesised that a high NLR is prognostic for an inferior OS in patients enrolled in a phase 1 trial. We aimed to integrate NLR into the RMH score in order to improve the discriminative ability of the model for OS.

## Methods

### Study design and patient eligibility

This retrospective study considered consecutive cancer patients referred to the Drug Development Unit, RMH, for consideration of a phase 1 trial between July 2004 and February 2014. The test cohort included 300 patients treated in a phase 1 trial, with a minimum of 40 patients with breast cancer, colorectal cancer, ovarian cancer, non-small cell lung cancer and prostate cancer. The validation cohort comprised of a further 1000 patients treated in a phase 1 trial, with no stratification for tumour type. Data were collected on age, gender, performance status, tumour type, date of first visit to Drug Development Unit, tumour type, therapeutic steroid use at new patient visit, date of first dose of investigational medicinal product and date of death or last follow-up. The following laboratory parameters were collected from the first visit: absolute neutrophil count, absolute lymphocyte count, LDH and albumin. The computerised tomography scan performed within 2 months of the first dose of the investigational medicinal product was used to assess the burden of disease. The RMH score was then calculated with one point ascribed to each of the following: albumin <35 g l^−1^, LDH>upper limit of normal, and >2 sites of metastases. Patients with an RMH score of 0–1 were compared with an RMH score of 2–3. The NLR was calculated with the absolute neutrophil count divided by absolute lymphocyte count. All patients included in this analysis had given their informed consent for participation in phase 1 trials approved by our Institutional Review Board, which also granted their approval for this analysis.

### Statistical considerations

An unpaired *t*-test and a one-way ANOVA were used to compare the association between prognostic factors with the NLR (Armitage *et al*, 2001). Given the variation in the optimal NLR thresholds for different tumour types, the NLR threshold was not prespecified. Instead, the NLR was stratified into quartiles based on the test cohort. The median OS was calculated for each quartile and quartile-1 was used as the reference category for comparing OS. The length of OS was calculated in months from the date of first dose of the investigational medicinal product to the date of death or last visit; patients who were alive or lost to follow-up at the date of last visit were censored. The potential binary confounders in the NLR's ability to predict for OS were determined in a univariate analysis using the Kaplan–Meier product-limit estimates (Armitage *et al*, 2001). As the NLR has a skewed distribution, the log-transformed NLR (Log_10_NLR) was used as a continuous variable. Other continuous variables were converted to binary variables using the following cutoffs: age <65 *vs* ⩾65 years, RMH score 0–1 *vs* 2–3, albumin <35 *vs* ⩾35 g l^−1^, LDH ⩽upper limit of normal *vs* >upper limit of normal, absolute neutrophil count ⩽5 × 10^9^ l^−1^ *vs* >5 × 10^9^ l^−1^, and absolute lymphocyte count <0.7 × 10^9^ l^−1^ *vs* ⩾0.7 × 10^9^ l^−1^. Variables that were associated with NLR were further analysed in a multivariate analysis using Cox proportional hazards model (Armitage *et al*, 2001). Furthermore, we analysed the binary outcomes of NLR25's, NLR50's and NLR75's ability to predict for OS in a multivariate analysis. Bonferroni correction for multiple comparisons was applied, with statistical significance defined as *P*<0.125 for Log_10_NLR, NLR25, NLR50 and NLR75.

Receiver operator characteristic (ROC) curve analysis was used to test the discriminative ability of the models combining the RMH score and NLR-measure (Hanley and Mcneil, 1983). Where the NLR-measure was binary, it was given a score of 0 when <binary cutoff defined by this analysis and a score of 1 if ⩾binary cutoff defined by this analysis. Harrell's concordance index (*C*-index) was used to rank the scores' ability to discriminate patients according to OS (Hanley and Mcneil, 1983). The *C*-indices were compared using the non-parametric paired method, based on correlated *U* statistics (two-sided test, with *α*=0.05) (Delong *et al*, 1988).

The model composed of the RMH score and the NLR-measure that produced the highest statistically significant *C*-index was assessed for its ability to associate with OS. Kaplan–Meier survival curves were constructed for the individual scores, and a binary scoring system was developed based on the clustering of the survival curves.

### Statistical software

Statistical analyses were performed using IBM SPSS Statistics, v22.0 (IBM Corp., Armonk, NY, USA), MedCalc, v12.7.1 (MedCalc Software, Ostend, Belgium) and GraphPad Prism v6.0 (GraphPad Software, La Jolla, CA, USA).

## Results

### Descriptive statistics

#### Descriptive statistics—test cohort

**Figure 1:**
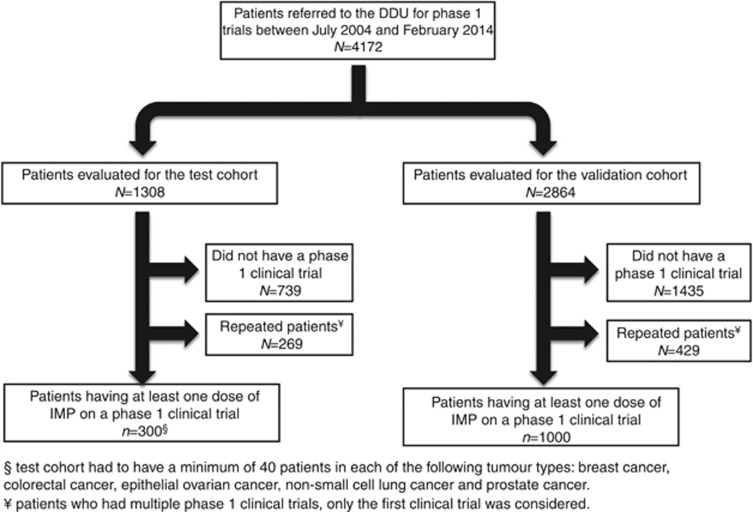
**Flow diagram illustrating patient disposition in the test cohort and the validation cohort.** Abbreviation: DDU=Drug Development Unit, Royal Marsden Hospital, UK.

Between July 2004 and February 2014, 4172 patients were considered for phase 1 trial at the Drug Development Unit. Of these, 1308 patients were reviewed for the test cohort, with 300 patients treated in a phase 1 trial (Figure 1). Of these patients, 15% had breast cancer, 13% had colorectal cancer, 13% had ovarian cancer, 13% had non-small cell lung cancer, 14% had prostate cancer and 31% had other tumour types. The performance status was ECOG 0 in 35%, and ECOG 1–2 in 65%. The RMH score was 0–1 in 66% and 2–3 in 34% of patients. The median age was 60 years (interquartile range (IQR) 48–67), and 47% were male. The median absolute neutrophil count was 4.24 × 10^9^ l^−1^ (IQR 3.06–5.68), and the median absolute lymphocyte count was 1.39 × 10^9^ l^−1^ (IQR 1.02–1.82). The median NLR was 3.08 (IQR 2.06–4.49; Table 1). Stratification for these parameters for the five main tumour types in the test cohort is summarised in Table 1. The median OS was 8.6 months (95% CI 7.4–10.1), with an event rate of 66% and a median follow-up of 6.9 months.

#### Descriptive statistics—validation cohort

Of the patients referred, 2864 patients were reviewed for the validation cohort, with 1000 patients treated in a phase 1 trial (Figure 1). Of these patients, 9% had breast cancer, 18% had colorectal cancer, 14% had ovarian cancer, 6% had non-small cell lung cancer, 8% had prostate cancer and 46% had other tumours. The performance status was ECOG 0 in 37%, and ECOG 1–2 in 63%. The RMH score was 0–1 in 74% and 2–3 in 26%. The median age was 58 years (IQR 49–65), and 48% were male. The median absolute neutrophil count was 4.20 × 10^9^ l^−1^ (IQR 3.21–5.70), and the median absolute lymphocyte count was 1.39 × 10^9^ l^−1^ (IQR 0.99–1.80). The median NLR was 3.11 (IQR 2.13–4.92). The distribution of baseline parameters were similar in the test and validation cohorts, with the exception of the validation cohort having higher haemoglobin (124 *vs* 122 g l^−1^, *P*=0.006) and the validation cohort having more RMH score 0 and the test cohort having more RMH score 2 patients (*P*=0.01; Table 1). The latter can be explained by more >2 sites of metastases in the test cohort (*P*<0.0001). The median OS was 8.8 months (95% CI 8.0–9.5), with an event rate of 85% and a median follow-up of 7.5 months.

### Association of NLR with baseline characteristics

#### Association of NLR with baseline characteristics—test cohort

The test cohort was analysed to determine the association of baseline characteristics with NLR (Supplementary Table S1). Therapeutic steroid use was associated with a higher NLR (5.5 *vs* 3.6, *P*=0.0002), as was a performance status of ECOG 1–2 (4.1 *vs* 3.3, *P*=0.04). Although an albumin <35 g l^−1^ was associated with a high NLR (5.9 *vs* 3.4, *P*=0.0007), there was no association between a high NLR and the RMH score. Non-small cell lung cancer had the highest NLR, with the lowest NLR in ovarian cancer; however, the differences in NLR between tumour types were not significant.

#### Association of NLR with baseline characteristics—validation cohort

The validation cohort was analysed to determine the association of baseline characteristics with NLR (Supplementary Table S1). Therapeutic steroid use (7.0 *vs* 4.0, *P*<0.0001), a performance status of ECOG 1-2 (4.8 *vs* 3.4, *P*<0.0001) and an RMH score of 2-3 (5.4 *vs* 3.9, *P*<0.0001) were associated with a higher NLR. The latter was driven by the presence of a low albumin (4.1 *vs* 6.8, *P*<0.0001). Again, non-small cell lung cancer patients had the highest NLR, with the lowest NLR being reported in ovarian cancer patients; however, the differences in NLR between the tumour types were not statistically significant.

### Prognostic value of NLR

#### Prognostic value of NLR—test cohort

**Figure 2:**
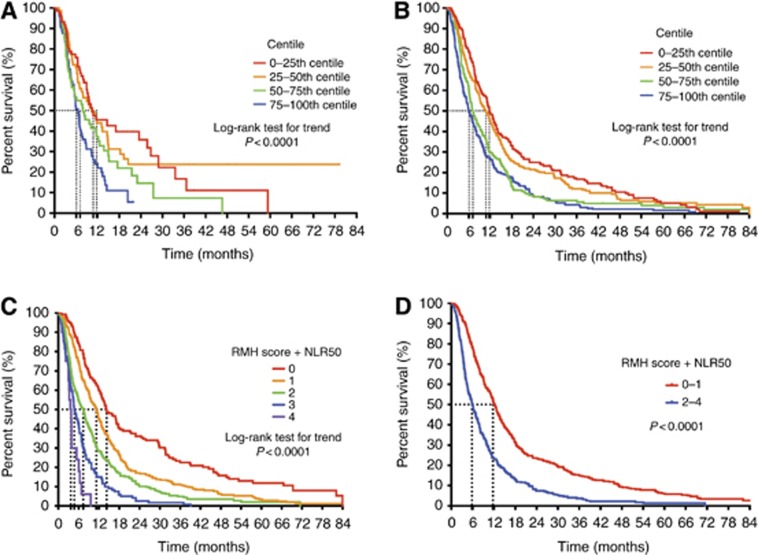
**Overall survival (OS) analysis for the NLR quartiles, using Kaplan–Meier product-limit estimates method.** The differences in OS were tested using the log-rank test. OS was analysed for the (**A**) test cohort and (**B**) validation cohort with the data stratified into the first, second, third and fourth quartiles. Both analyses show lower quartiles having a better survival when compared with the higher quartiles, with a statistically significant for trend. The ‘RMH score+NLR50' model for OS (**C**) with the discrete scores of 0, 1, 2, 3 and 4 and (**D**) with the clustering of scores 0–1 and 2–4, all for the validation cohort. Abbreviations: NLR=neutrophil–lymphocyte ratio; OS=overall survival.

To determine the prognostic value of the NLR, OS was assessed in each of the NLR quartiles, with quartile-1 being used as a reference group. The median OS was 10.5 months (95% CI 8.9–23.5) for quartile-1, 10.3 months (95% CI 7.0–14.7) for quartile-2 (HR=1.04, *P*=0.8), 7.9 months (95% CI 4.8–11.6) for quartile-3 (HR=1.56, *P*=0.03) and 6.5 months (95% CI 5.4–7.6) for quartile-4 (HR=2.08, *P*=0.001; *P*-value for trend *P*<0.0001; Figure 2A).

To further demonstrate the prognostic ability of the NLR for OS, we analysed the binary outcomes NLR25, NLR50 and NLR 75. The median OS for NLR25 was 10.5 *vs* 7.6 months (HR=1.26, *P*=0.2); for NLR50 was 10.5 *vs* 6.8 months (HR=1.69, *P*=0.0001); and for NLR75 was 9.7 *vs* 6.5 months (HR=1.78, *P*=0.0001).

#### Prognostic value of NLR—validation cohort

In the validation cohort, median OS was 11.7 months (95% CI 10.9–13.5) for quartile-1, 10.8 months (95% CI 9.0–12.4) for quartile-2 (HR=1.13, *P*=0.2), 7.1 months (95% CI 6.3–8.8) for quartile-3 (HR=1.60, *P*<0.0001) and 6.1 months (95% CI 8.0–9.5) for quartile-4 (HR=1.85, *P*<0.0001; *P*-value for trend *P*<0.0001; Figure 2B).

The median OS for NLR25 was 11.7 *vs* 7.9 months (HR=1.47, *P*<0.0001); for NLR50 was 11.4 *vs* 6.7 months (HR=1.62, *P*<0.0001); and for NLR75 was 9.9 *vs* 6.1 months (HR=1.57, *P*<0.0001).

### Univariate analysis/multivariate analysis for OS

#### Univariate analysis/multivariate analysis for OS—test cohort

A univariate analysis and multivariate analysis were used for the analysis of OS in the test cohort (Table 2A). Univariate analysis identified an RMH score of 2–3 (HR=0.55, *P*<0.0001), a performance status of ECOG 1–2 (HR=0.62, *P*=0.002) and an absolute neutrophil count >5 × 10^9^ l^−1^l (HR=0.65, *P*=0.003) as associating with poor OS. Interestingly, a low absolute lymphocyte count did not associate with OS (*P*=0.3). All individual components of the RMH score were significantly associated with a worse survival. Importantly, therapeutic steroid use did not associate with OS (*P*=0.09).

A multivariate analysis was used to analyse the impact of potential confounders on the prognostic ability of the Log_10_NLR for OS. The RMH score, performance status and absolute neutrophil count were used as potential confounders in the model, as identified in the univariate analysis, with the addition of tumour type. The Log_10_NLR remained significantly associated with OS (adjusted HR=2.22, *P*=0.002). Similar results were seen when the multivariate analysis was modeled for NLR50 (adjusted HR=0.68, *P*=0.01) and NLR75 (adjusted HR=0.69, *P*=0.04); however, these did not remain significantly associated after applying the Bonferroni correction. As the NLR25 was not statistically significant in the univariate analysis, this was not analysed further. The interaction test did not identify an interaction between the RMH score and the Log_10_NLR (*P*=0.9).

#### Univariate analysis/multivariate analysis for OS—validation cohort

A univariate analysis and multivariate analysis were used for the analysis of OS in the validation cohort (Table 2B). The univariate analysis identified an RMH score of 2–3 (HR=0.51, *P*<0.0001), a performance status of ECOG 1-2 (HR=0.77, *P*=0.0002), an absolute neutrophil count >5 × 10^9^ l^−1^ (HR=0.62, *P*<0.0001) and an absolute lymphocyte count <0.7 × 10^9^ l^−1^ (HR=1.29, *P*<0.0001), as associating with poor OS. All components of the RMH score remained significantly associated with an inferior OS. Therapeutic steroid use was not a confounding factor (*P*=0.1).

The RMH score, performance status, absolute neutrophil count and absolute lymphocyte count were used as potential confounders in the multivariate analysis, as identified in the univariate analysis, with the addition of tumour type. The Log_10_NLR remained a significant prognostic factor (adjusted HR=2.12, *P*<0.0001), as did NLR50 (adjusted HR=1.37, *P*=0.0002). Similar results were seen when the multivariate analysis was modeled for NLR25 (adjusted HR=1.22, *P*=0.02) and NLR75 (adjusted HR=1.25, *P*=0.02); however, again these were not significantly associated with OS after applying the Bonferroni correction. The interaction test did not identify an interaction between the RMH score and the Log_10_NLR (*P*=0.1).

### ROC curve analysis for integrating NLR and RMH score

#### ROC curve analysis for integrating NLR and RMH score—test cohort

**Figure 3:**
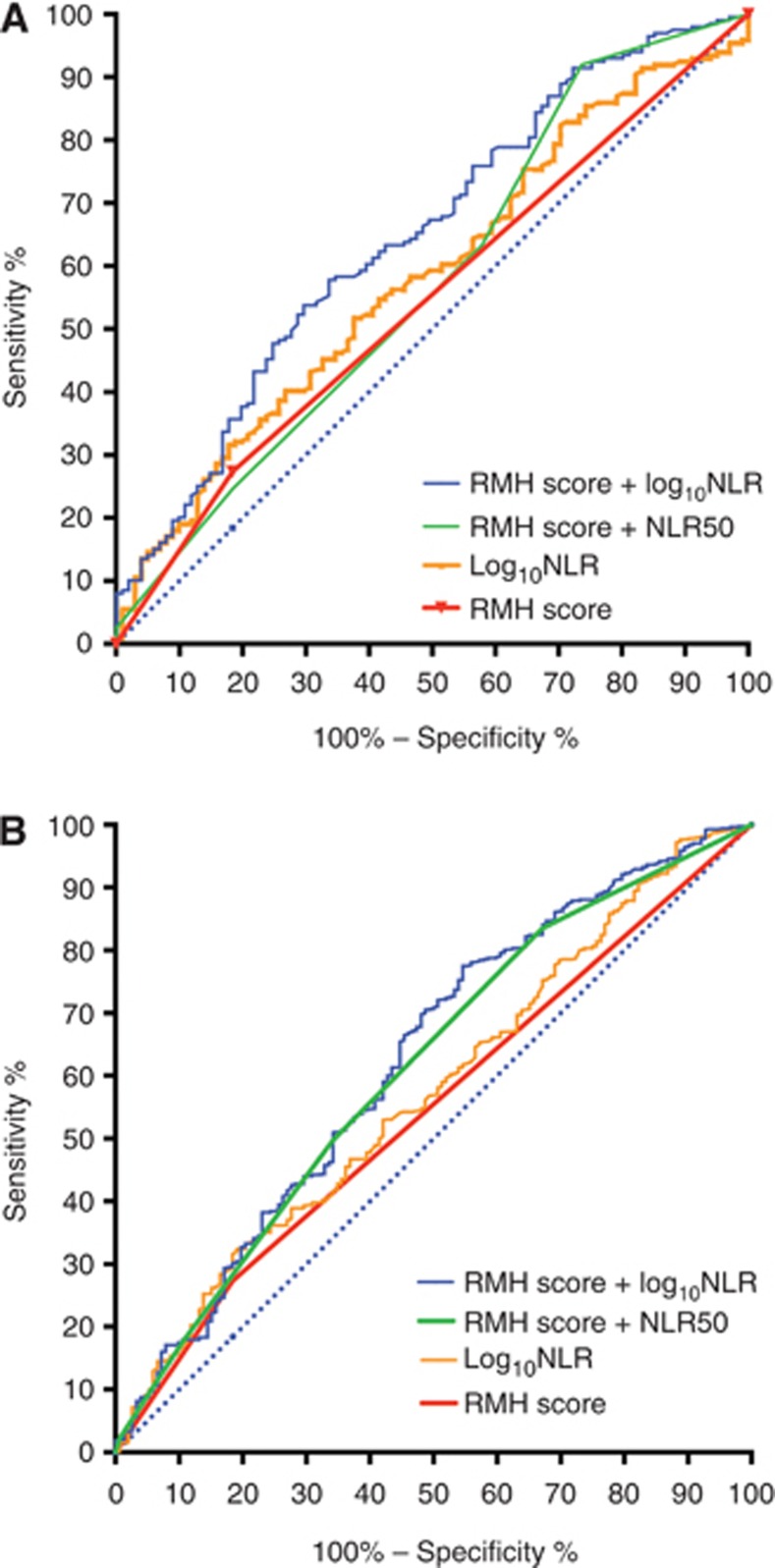
**Receiver operator characteristic curve analysis for the test and validation cohorts.** The receiver operator characteristic curve analysis for the (**A**) test and (**B**) validation cohorts shows the ability of the different models to discriminate for overall survival. The models tested in addition to the RMH score alone and the NLR alone were RMH score+NLR50 and RMH score+Log_10_NLR, as they were found to be significantly associated with overall survival in multivariate analysis. Abbreviations: NLR=Neutrophil–lymphocyte ratio; RMH=Royal Marsden Hospital Score.

ROC curve analysis assessed the ability of the NLR to discriminate for OS compared with the RMH score and to evaluate any improvement of its discriminative ability by adding an NLR-measure. The RMH score+Log_10_NLR and RMH score+NLR50 were evaluated here (Figure 3A and Table 3A). The *C*-index for the RMH score alone was 0.63 (95% CI 0.56–0.70; *P*=0.0002) and for the Log_10_NLR alone was 0.58 (95% CI 0.52–0.65; *P*=0.02), with no difference in the discriminating ability between them (*P*=0.9). Both models tested were significant in discriminating for OS. Comparing these to the RMH score alone showed that RMH score+Log_10_NLR (*P*=0.005) significantly improved the discriminative ability of the model; however, the RMH score+NLR50 did not (*P*=0.006; Table 2A).

#### ROC curve analysis for integrating NLR and RMH score—validation cohort

ROC curve analysis was performed to assess the ability of the NLR to discriminate for OS in the validation cohort, as described above (Figure 3B and Table 3B). The *C*-index for the RMH score alone was 0.55 (95% CI: 0.56–0.70; *P*<0.0001) and for the Log_10_NLR alone was 0.57 (95% CI 0.52–0.62; *P*=0.004), with no difference in the discriminating ability between them (*P*=0.3). Both models tested were significant in discriminating for OS. The RMH score+NLR50 model, a model that resulted in a discrete score, had the highest *C*-index (*C*-index 0.61, 95% CI 0.56–0.66, *P*<0.0001).

#### Prognostic ability of the RMH score+NLR50 model

The prognostic power of the RMH score+NLR50 model was analysed for OS (Figure 2C). The discrete scores from this model were 0, 1, 2, 3 or 4, with the median OS being 14.6, 11.2, 7.4, 4.7 and 3.7 months, respectively (*P*<0.0001 for trend). Scores 0–1 and scores 2–4 clustered together (Figure 2C), with a median OS of 12.2 *vs* 6.0 months, respectively (HR=0.51, *P*<0.0001; Figure 2D).

## Discussion

Inflammation has a critical role in tumorigenesis. The NLR is a marker of inflammation that is readily derived from the peripheral blood. The work presented here is the first study to validate the prognostic significance of the NLR in a large cohort of phase 1 clinical trial patients, demonstrating a 5.6-month significant difference in OS between quartile-1 and quartile-4, and a negative linear relationship between the HR and the NLR, indicating that the higher the NLR the worse the prognosis. The HR remained significant, after adjusting for the RMH score, performance status and absolute neutrophil count. Given the strong prognostic association, we were able to integrate the RMH score and the NLR-measure, improving on the discriminating ability of the RMH score for OS.

Both the RMH score+Log10NLR and the RMH score+NLR50 were highly significant for improving the model's discriminative ability for OS. We would favour the RMH score+NLR50 model, as it had the highest significant *C*-index for OS. It is also a practical model to use in the clinic for the selection of patients for phase 1 clinical trials in that the model produces discrete scores, with scores of 0–1 having a median OS of 12.2 months and scores of 2–4 having a median OS of 6.0 months. Our data would suggest that the optimal NLR threshold in a phase 1 population is 3.0.

The utility of the RMH score+NLR50 model lies in the selection of patients for participation in phase 1 clinical trials. As the eligibility criteria for most phase 1 clinical trials stipulate that patients should have a life expectancy of >3 months, patients with an RMH score+NLR50 score of 0–1 can certainly be considered. However, caution should be exercised in patients with an RMH score+NLR50 score of 2–4, as some of these patients will have a survival measured between 3 and 6 months. Second, the discriminating ability of the NLR alone was the same as that of the RMH score alone, suggesting that the NLR could be used instead of the RMH score in assessing a new patient for consideration of a phase 1 clinical trial, particularly when an up-to-date computerised tomography scan is not available. Although the interaction test between NLR and RMH score was negative, biologically, it is conceivable that there may be a potential interaction, as suggested by the RMH score 2–3 having a significantly higher NLR compared with RMH score 0–1. Hypoalbuminaemia is an independent biomarker of tumour inflammation and poor prognosis (Mcmillan, 2013), as is a raised LDH (Agarwala *et al*, 2009), both being crucial components of the RMH score. It is noteworthy that C-reactive protein levels are prognostic in cancer, as demonstrated by the Glasgow Prognostic Score; however, this has not been evaluated in a phase 1 patient population (Mcmillan, 2013) and deserves further consideration.

The NLR has potential application in drug development. The mapping of the human kinome has led to accelerated drug discovery and personalised medicine. This has been paralleled with biomarker development, in order to enrich trials with patients more likely to respond, including phase 1 trials. Current paradigms in trial design rely on genomic biomarkers, based on gene amplification or loss, or genetic mutations (Carden *et al*, 2010; Bauer *et al*, 2014). Biomarkers predictive of response to immunotherapies remain an area of unmet need. This work has validated the NLR as a prognostic biomarker in phase 1 trial patients, identifying patients whose tumours are generating an inflammatory response. There is scope for further investigation of NLR as a predictive biomarker of response to immunotherapies, particularly with immune checkpoint targeting drugs such as CTLA-4 and PD-1/PDL-1 targeting antibodies, and the utility of normalisation of the NLR with treatment (Pinato *et al*, 2014).

The biology underlying the role of inflammation in cancer pathogenesis and progression is an area of intense research. A raised NLR is a result of a high absolute neutrophil count and/or a low absolute lymphocyte count. Our univariate analysis showed that a raised absolute neutrophil count was significantly associated with poor OS, compared with a low absolute lymphocyte count. Tumour-associated neutrophils, defined as having CD11b^+^/Gr-1^+^ expression, have been recognised as being a poor prognostic factor (Fridlender and Albelda, 2012). Patients with tumour-associated neutrophils have a raised absolute neutrophil count in the peripheral blood (Schmidt *et al*, 2005). This concept lends itself to two potential therapeutic opportunities. First, two phenotypes of tumour-associated neutrophils have been recognised; the N1-phenotype resulting from low TGF*β*/high IFN*β*, causing tumour growth retardation; and the N2-phenotype resulting from high TGF*β*/low IFN*β*, causing tumour growth. Depletion of TGF*β* can shift the phenotype towards N1, causing growth retardation (Fridlender *et al*, 2009). Second, murine mammary adenocarcinoma models have shown that neutrophil depletion with anti-granulocyte receptor-1 antibody can result in tumour regression (Pekarek *et al*, 1995). Di Mitri *et al* (2014) have shown in PTEN-null prostate tumours in mice that CD11b^+^/Gr-1^+^ myeloid cells prevent tumour senescence through secretion of IL-1RA and that CD11b^+^/Gr-1^+^ myeloid cells can be reduced using a CXCR2 antagonist, encouraging tumour senescence following docetaxel.

Several validated prognostic models have been developed for patients referred for phase 1 clinical trials. The work by Pinato *et al* (2014) is the only model to take inflammation into account. However, in contrast to this work, the merits of our data are that it has been validated in a large sample size. Moreover, the NLR was analysed as a continuous variable in order to maintain statistical power. We deliberately did not prespecify an NLR threshold but subdivided our population into quartiles in an attempt to optimise this statistical evaluation. Our results add to the established RMH score, improving on the prognostic model for patient selection onto phase 1 trials. This is the first publication to define the optimal NLR in a phase 1 patient population.

Limitations of this study include that it is a single institution retrospective analysis. Further prospective multicenter validation should be now considered in an external data set. The results presented here are from patients treated in phase 1 trials with cytotoxic chemotherapy and/or small-molecule inhibitors, making the data difficult to extrapolate to patient being treated with immunotherapies. Validation in this specific subpopulation receiving immunotherapies is required.

The NLR may be an objective measure of inflammation that can be easily derived from routine laboratory assessments, in addition to the RMH score. The NLR has been validated as a prognostic tool for OS in patients being treated in a phase 1 trial. Using the NLR of 3.0 in our 1000 patient validation cohort, the RMH score+NLR50 generated the most prognostic dichotomisation of the population for OS by 6.2 months. This robust prognostic biomarker must now be evaluated as a predictive and response biomarker for cancer immunotherapies.

## Figures and Tables

**Table 1:** Baseline descriptive statistics for the 300 patients analysed in the test cohort and the 1000 patients analysed in the validation cohort

	**Test cohort**								
	**Breast cancer**	**Colorectal cancer**	**Ovarian cancer**	**Non-small cell lung cancer**	**Prostate cancer**	**Other tumour types**	**All patients in the test cohort**	**Validation cohort**	**Comparison of all patients in the test and validation cohorts** ***P*-value**^a^
Number of patients	46 (15%)	40 (13%)	40 (13%)	40 (13%)	41 (14%)	93 (31%)	300 (100%)	1000 (100%)	—
**ECOG**									
0	22	13	18	9	12	31	105 (35%)	370 (37%)	0.06
1	24	26	22	31	19	72	194 (64%)	607 (60·7%)	
2	0	1	0	0	0	0	1 (0·3%)	23 (2·3%)	
**RMH score**									
0	10	7	14	8	4	27	70 (23%)	319 (31·9%)	0.01*
1	20	12	18	22	21	36	129 (43%)	421 (42·1%)	
2	14	21	7	10	15	26	93(31%)	235 (23·5%)	
3	2	0	1	0	1	4	8 (3%)	25 (2·5%)	
Gender—male	0%	63%	0%	60%	100%	55%	141 (47%)	484 (48%)	0.7
Median age (IQR)	51 (46–62)	61 (47–67)	58 (47–64)	62 (56–70)	66 (61–74)	56 (45–67)	60 (48–67)	58 (49–65)	0.1
Median haemoglobin (IQR)	123 (118–134)	131 (117–143)	114 (107–127)	122 (113–134)	124 (114–134)	119 (108–131)	122 (111–133)	124 (114–135)	0.006**
Median platelets (IQR)	240 (208–319)	201 (144–266)	292 (215–357)	297 (234–376)	224 (195–285)	256 (194–353)	245 (197–330)	259 (208–319)	0.8
Median white cell count (IQR)	5.8 (4.7–7.5)	7.0 (6.1–8.4)	5.3 (4.5–7.1)	8.1 (6.0–6.8)	6.5 (5.5–8.4)	6.5 (5.3–8.4)	6.5 (5.2–8.3)	6.6 (5.3–8.2)	0.5
Median ANC (IQR)	3.65 (2.61–4.83)	4.31 (3.46–5.82)	3·30(2.52–5.04)	5.68 (3.79–6.82)	3.96 (3.15–5.78)	4.38 (3.25–5.94)	4.24 (3.06–5.68)	4.2 (3.21–5.70)	0.7
Median ALC (IQR)	1.52 (1.08–1.91)	1.48 (1.07–1.99)	1.42 (1.08–1.76)	1.40 (1.20–2.12)	1.29 (0.94–1.83)	1.28 (0.95–1.74)	1.39 (1.02–1.82)	1.30 (0.99–1.80)	0.2
Median LDH (IQR)	174 (143–283)	204 (160–260)	166 (143–228)	161 (129–223)	178 (153–220)	177 (132–231)	178 (143–232)	171 (140–224)	0.8
Median albumin (IQR)	39 (36–41)	37 (35–39)	36 (34–39)	35 (33–37)	36 (34–38)	36 (33–38)	37 (34–39)	36 (33–39)	0.07
Greater than two sites of metastases	33 (72%)	28 (70%)	21 (53%)	26 (65%)	37 (90%)	42 (45%)	196 (65%)	497 (49·7%)	<0.0001***
Median NLR (IQR)	2.38 (1.72–3.42)	3.30 (2.20–4.45)	2.51 (1.70–3.17)	3.96 (2.28–6.20)	3.07 (2.20–4.81)	3.45 (2.17–5.07)	3.08 (2.06–4.49)	3.11 (2.13–4.92)	0.06

Abbreviations: ALC=absolute leukocyte count; ANC=absolute neutrophil count; ECOG=Eastern Cooperative Oncology Group; IQR=interquartile range; LDH=lactate dehydrogenase; NLR=neutrophil–lymphocyte ratio; RMH=Royal Marsden Hospital.

The test cohort data has been stratified according to the five major tumour types of breast cancer, colorectal cancer, ovarian cancer, non-small cell lung cancer and prostate cancer. Continuous variables have been presented as the median value and the interquartile range.

a Categorical data were compared using Chi-squared test and continuous data were compared using unpaired *t*-test. *P<0.05, **P<0.01, ***P<0.001.

**Table 2:** Univariate and multivariate analysis for the potential prognostic factors associated with overall survival

		**Overall survival**			
		**Univariate model**		**Multivariate model**	
**Variable**	**N**	**HR (95% CI)**	***P*-value**	**HR (95% CI)**	***P*-value**
**(A) Test cohort**					
Age (<65 *vs* ⩾65 years)	202/98	0.92 (0.69–1.24)	0.6	—	—
Gender (male *vs* female)	159/141	0.85 (0.65–1.13)	0.3	—	—
Steroids (yes *vs* no)	37/263	0.72 (0.50–1.06)	0.09	—	—
RMH score (0–1 *vs* 2–3)	199/101	0.55 (0.37–0.69)	<0.0001***	0.59 (0.44–0.80)	0.0005***
Albumin (<35 *vs* ⩾35 g l^−1^)	18/282	0.30 (0.14–0.62)	0.001**	—	—
LDH (⩽ULN *vs* >ULN)	175/125	0.53 (0.39–0.71)	<0.0001***	—	—
Sites of metastases (⩽2 *vs* >2)	104/196	0.69 (0.52–0.92)	0.01*	—	—
Performance status (ECOG 0 *vs* ECOG 1–2)	106/194	0.62 (0.48–0.84)	0.002**	0.72 (0.53–0.98)	0.04*
Absolute neutrophil count (⩽5 × 10^9^ l^−1^ *vs* >5 × 10^9^ l^−1^)	193/107	0.65 (0.46–0.85)	0.003**	1.31 (0.95–1.81)	0.1
Absolute lymphocyte count (<0.7 × 10^9^ l^−1^ *vs* ⩾0.7 × 10^9^ l^−1^)	23/277	1.34 (0.78–2.50)	0.3	—	—
Tumour type	300	—	—	0.98 (0.91–1.06)	0.6
Log_10_NLR	300	—	—	2.22 (1.17–4.23)	0.002**
NLR25 (NLR ⩽2.06 *vs* NLR >2.06)	73/227	1.26 (0.91–1.71)	0.2	—	—
NLR50 (NLR ⩽3.08 *vs* NLR >3.08)	151/149	1.69 (1.31–2.31)	0.0001***	06.8 (0.49–0.92)	0.004**
NLR75 (NLR ⩽4.45 *vs* NLR >4.45)	224/76	1.78 (1.41–2.87)	0.0001***	0.69 (0.48–0.98)	0.04
**(B) Validation cohort**					
Age (<65 *vs* ⩾65 years)	751/249	1.11 (0.95–1.30)	0.6	—	—
Gender (male *vs* female)	484/516	0.85 (0.74–0.97)	0.2	—	—
Steroids (yes *vs* no)	108/892	1.23 (0.96–1.56)	0.1	—	—
RMH score (0–1 *vs* 2–3)	740/260	0.51 (0.36–0.51)	<0.0001***	1.82 (1.56–2.14)	<0.0001***
Albumin (<35 *vs* ⩾35 g l^−1^)	81/919	0.47 (0.24–0.47)	<0.0001***	—	—
LDH (⩽ULN *vs* >ULN)	612/388	0.57 (0.46–0.62)	<0.0001***	—	—
Sites of metastases (⩽2 *vs* >2)	503/497	0.67 (0.58–0.76)	<0.0001***	—	—
Performance status (ECOG 0 *vs* ECOG 1–2)	370/630	0.77 (0.67–0.88)	0.0002***	1.23 (1.07–1.42)	0.005**
Absolute neutrophil count (⩽5 × 10^9^ l^−1^ *vs* >5 × 10^9^ l^−1^)	641/359	0.62 (0.50–0.68)	<0.0001***	1.37 (1.17–1.60)	0.0001***
Absolute lymphocyte count (<0.7 × 10^9^ l^−1^ *vs* ⩾0.7 × 10^9^ l^−1^)	91/909	1.29 (1.03–1.74)	0.03*	0.78 (0.57–1.07)	0.1
Tumour type	1000	—	—	0.98 (0.94–1.01)	0.3
Log_10_NLR	1000	—	—	2.12 (1.50–2.99)	<0.0001***
NLR25 (NLR ⩽2.06 *vs* NLR >2.06)	235/765	1.47 (1.23–1.65)	<0.0001***	1.22 (1.03–1.45)	0.02
NLR50 (NLR ⩽3.08 *vs* NLR >3.08)	497/503	1.62 (1.45–1.91)	<0.0001***	1.37 (1.16–1.60)	0.0002***
NLR75 (NLR ⩽4.45 *vs* NLR >4.45)	709/291	1.57 (1.42–1.97)	<0.0001***	1.25 (1.04–1.51)	0.02

Abbreviations: CI=confidence interval; ECOG=Eastern Cooperative Oncology Group; HR=hazard ratio; LDH=lactate dehydrogenase; NLR=neutrophil–lymphocyte ratio; RMH=Royal Marsden Hospital; ULN=upper limit of normal.

The results for the test cohort are shown in Table 2A and the results for the validation cohort are shown in Table 2B. Only variables that were found to be associated with overall survival in the univariate model were analysed in the multivariate model, in addition to the tumour type and Log_10_NLR. Bonferroni correction for multiple comparisons was applied, with statistical significance defined as *P*<0.125 for Log10NLR, NLR25, NLR50 and NLR75. *, **, ***Statistically significant.

**Table 3:** Results for the receiver operator characteristic curve analysis for the test cohort in (A) and the validation cohort in (B), summarising the *C*-indices, the 95% confidence interval for the *C*-indices and the *P*-value for the discriminative ability

	**Area under curve (C-index)**	**95% confidence interval**	**P-value**	**Pairwise comparison to RMH model, P-value**
**(A) Test cohort**				
—RMH score	0.630	0.56–0.70	0.0002***	—
—Log_10_NLR	0.583	0.52–0.65	0.02*	0.9
—RMH score+NLR50	0.583	0.57–0.70	0.0002***	0.06
—RMH score+Log_10_NLR	0.647	0.58–0.71	<0.0001***	0.005**
**(B) Validation cohort**				
—RMH score	0.545	0.56–0.65	<0.0001***	—
—Log_10_NLR	0.573	0.52–0.62	0.004**	0.3
—RMH score+NLR50	0.611	0.56–0.66	<0.0001***	<0.0001***
—RMH score+Log_10_NLR	0.623	0.57–0.67	<0.0001***	<0.0001***

Abbreviations: NLR=neutrophil–lymphocyte ratio; RMH=Royal Marsden Hospital.

The last column shows the non-parametric paired comparison of the *C*-indices to the RMH score. *P<0.05, **P<0.01, ***P<0.001.
