# Supplementary material for: The neutrophil–lymphocyte ratio and its utilisation for the management of cancer patients in early clinical trials
Source: Br J Cancer. 2015 Feb 26;112(7):1157–65. doi: 10.1038/bjc.2015.67 (PMC4385959; doi:10.1038/bjc.2015.67)
Supplement: Supplementary Information [file bjc201567x1.doc]

|  | **Test Cohort** | | **Validation Cohort** | |
| --- | --- | --- | --- | --- |
|  | **Mean NLR** | **p-value (Unpaired t-test)** | **Mean NLR** | **p-value (Unpaired t-test)** |
| **Steroids** |  |  |  |  |
| Steroid Use | 5·5 | 0·0002 *** | 7·0 | <0·0001 *** |
| No Steroid Use | 3·6 |  | 4·0 |  |
| **Gender** |  |  |  |  |
| Male | 4·1 | 0·09 | 4·5 | 0·2 |
| Female | 3·5 |  | 4·1 |  |
| **Tumour Type** |  |  |  |  |
| Breast cancer | 3·5 | 0·2 **^¶^** | 4·1 | 0·5 **^¶^** |
| Colorectal cancer | 3·6 |  | 4·3 |  |
| Ovarian Cancer | 2·9 |  | 3·8 |  |
| Non-Small Cell Lung Cancer | 4·4 |  | 4·9 |  |
| Prostate Cancer | 4·0 |  | 4·0 |  |
| Other | 4·1 |  | 4·4 |  |
| **Performance Status** |  |  |  |  |
| ECOG 0 | 3·3 | 0·04 * | 3·4 | <0·0001 *** |
| ECOG 1-2 | 4·1 |  | 4·8 |  |
| **RMH score** |  |  |  |  |
| RMH score 0-1 | 3·9 | 0·6 | 3·9 | <0·0001 *** |
| RMH score 2-3 | 3·7 |  | 5·4 |  |
| Albumin ≥35 g/L | 3·4 | 0·0007 *** | 4·1 | <0·0001 *** |
| Albumin <35 g/L | 5·9 |  | 6·8 |  |
| LDH >ULN | 4·2 | 0·07 | 4·7 | 0·01 * |
| LDH ≤ULN | 3·5 |  | 4·0 |  |
| Sites of metastases >2 | 3·8 | 0·7 | 4·7 | 0·01 * |
| Sites of metastases ≤2 | 3·9 |  | 4·0 |  |
| **¶** One way ANOVA | | | | |

**Supplementary Table 1:** NLR associations in the test cohort and validation cohort for baseline characteristics.

**Abbreviations**: *, *** - statistically significant; ECOG – Eastern Cooperative Oncology Group; RMH – Royal Marsden Hospital; LDH – lactate dehydrogenase; ULN – upper limit of normal.
